# The CRL4^VPRBP(DCAF1)^ E3 ubiquitin ligase directs constitutive RAG1 degradation in a non-lymphoid cell line

**DOI:** 10.1371/journal.pone.0258683

**Published:** 2021-10-14

**Authors:** N. Max Schabla, Patrick C. Swanson

**Affiliations:** Department of Medical Microbiology and Immunology, Creighton University, Omaha, Nebraska, United States of America; Chang Gung University, TAIWAN

## Abstract

The development of B and T lymphocytes critically depends on RAG1/2 endonuclease activity to mediate antigen receptor gene assembly by V(D)J recombination. Although control of RAG1/2 activity through cell cycle- and ubiquitin-dependent degradation of RAG2 has been studied in detail, relatively little is known about mechanisms regulating RAG1 stability. We recently demonstrated that VprBP/DCAF1, a substrate adaptor for the CRL4 E3 ubiquitin ligase complex, is required to maintain physiological levels of RAG1 protein in murine B cells by facilitating RAG1 turnover. Loss of VprBP/DCAF1 *in vivo* results in elevated RAG1 expression, excessive V(D)J recombination, and immunoglobulin light chain repertoire skewing. Here we show that RAG1 is constitutively degraded when ectopically expressed in a human fibroblast cell line. Consistent with our findings in murine B cells, RAG1 turnover under these conditions is sensitive to loss of VprBP, as well as CRL4 or proteasome inhibition. Further evidence indicates that RAG1 degradation is ubiquitin-dependent and that RAG1 association with the CRL4^VPRBP/DCAF1^ complex is independent of CUL4 activation status. Taken together, these findings suggest V(D)J recombination co-opts an evolutionarily conserved and constitutively active mechanism to ensure rapid RAG1 turnover to restrain excessive RAG activity.

## Introduction

During their differentiation from hematopoietic stem cells, B and T lymphocytes assemble antigen receptor genes from arrays of Variable, Diverse, and Joining segments through a process termed V(D)J recombination. This process requires the recombination activating gene proteins (RAG1 and RAG2), which together initiate V(D)J recombination by catalyzing the formation of DNA double-strand breaks (DBSs) at sites immediately adjacent to antigen receptor gene segments. The DNA ends are then processed and re-ligated by the non-homologous end-joining pathway to form functional immunoglobulin (Ig) or T cell receptor variable genes in B and T lymphocytes, respectively.

Structure-function analysis of the full-length (FL) RAG proteins has led to their division into ‘core’ (c) and ‘non-core’ (nc) regions, which are essential and dispensable for the catalytic activity of the RAG complex, respectively [1–4]. Auxiliary roles for the nc parts of both RAG proteins have been described. For ncRAG2 (spanning residues 352–527), phosphorylation of Thr490 stimulates polyubiquitylation by the SCF^Skp2^ E3 ubiquitin ligase and degradation of RAG2 at the G1/S phase boundary, effectively restricting V(D)J recombination to G0/G1 [5, 6]. Furthermore, a plant homeodomain located in ncRAG2 plays an essential role in genomic targeting of the RAG complex via its interaction with H3K4me3 [7, 8].

NcRAG1 includes the N-terminal third of RAG1 (1–383) as well as the C-terminal 31 residues (1009–1040). Though dispensable for catalytic activity, the N-terminal portion of ncRAG1 (N-ncRAG1) is required for efficient V(D)J recombination, as cRAG1 knock-in mice exhibit impaired rearrangement of Ig and T cell receptor genes, as well as reduced B and T cell numbers in peripheral lymphoid organs [9, 10]. Interestingly, ncRAG1 contains a functional Really Interesting New Gene (RING) E3 ubiquitin ligase domain [11]. Ubiquitin (Ub) is a small (~11 kDa) protein that is covalently attached to substrate proteins via a mechanism incorporating three classes of enzymes: E1 (Ub activating enzyme [UAE]), which catalyzes the charging of an E2 (Ub conjugating) with a Ub monomer, and E3 (Ub ligase), which facilitates transfer of the Ub from the E2 to a lysine residue of the substrate [12]. ncRAG1 is reported to catalyze the ubiquitylation of several substrates *in vitro*, including histone H3 [13, 14], KPNA1 [15], and itself [16, 17], although the physiological role of this activity is not fully understood. However, a P326G mutation in the RAG1 RING E3 ligase domain was recently shown to reduce V(D)J recombination efficiency and modestly increase RAG1 protein levels *in vivo*, establishing a clear regulatory role for this domain in V(D)J recombination [18].

The Schatz group has recently identified a nucleolar localization signal (NoLS) motif spanning aa243-249 of N-ncRAG1 [19]. They showed that a B cell line expressing a NoLS-deficient form of RAG1 exhibited increased V(D)J recombination. Furthermore, treating actively cycling mouse bone marrow-derived B cell progenitors with actinomycin D to disrupt nucleolar morphology increased DNA cleavage at the *Ig kappa* locus. These results, taken together, suggest that nucleolar localization of RAG1, along with cell cycle-dependent degradation of RAG2, helps restrict V(D)J recombination to G0/G1 [19].

An additional role implicated for N-ncRAG1 is as a protein-protein interaction module which serves to recruit accessory factors to regulate V(D)J recombination. Several proteins that bind N-ncRAG1 have been identified over the years [20–24], but in most cases, the biological significance of the association remains unclear. Our laboratory identified an interaction between ncRAG1 and Viral protein r Binding Protein (VprBP), a substrate adaptor subunit for the cullin (CUL) 4 RING-type E3 Ub ligase (CRL4) [20].

CRLs are modular E3 Ub ligase complexes in which substrate recruitment and catalytic functions are delegated to separate subunits, organized around one of seven CUL scaffold proteins [25]. CRL4 incorporates either one of two CUL4 paralogs (CUL4A or CUL4B), DNA Damage-Binding Protein 1 (DDB1), which adapts VprBP to CUL4A, and the small catalytic RING domain-containing protein Rbx1. CRL activity is positively regulated by covalent modification of the CUL protein with the small Ub-like protein (UBL) NEDD8 [26, 27]. The requirement for NEDD8 attachment to the CUL protein to activate the CRL has been exploited for global inhibition of CRLs by MLN4924 (Soucy et al., 2009), an inhibitor of NEDD8-activating enzyme (NAE), that initiates NEDD8 conjugation. VprBP is the founding member of a family of DDB1 and CUL4-associated factors identified as adaptors to the CRL4 E3 Ub ligase; thus, VprBP is also called DCAF1 (for review, see [28]). For simplicity, and based on our previous work, we will refer to VprBP henceforth.

Conditional inactivation of *Vprbp* during early B cell development *in vivo* causes a severe block at the pro- to pre-B cell transition, which is partially rescued by expression of an Eμ-BCL2 transgene [20, 29]. Interestingly, most B cells developing on the VprBP-deficient, BCL2-transgenic background express Igλ light chain-containing BCRs, rather than the normally predominating Igκ light chain [29]. This outcome is likely due to a transcription-independent increase in RAG1 protein levels in the absence of VprBP, leading to excessive kappa deletion and inactivation of the *Igk* locus [30]. Our further studies showed that loss of VprBP, or inhibiting CRL or proteasome activity, extended the half-life of RAG1, suggesting that CRL4^VprBP^ normally functions to target RAG1 to the proteasome for degradation [30]. Moreover, RAG1 degradation occurred in G0/G1-arrested B cells, in which RAG2 is stable, further establishing that RAG1 turnover relies on a mechanism which is distinct from the one responsible for degrading RAG2 [30]. Interestingly, Brecht *et al*. (2020) found that a RAG1 truncation mutant lacking aa1-215 (which disrupts association with CRL4^VprBP^ [20]) showed impaired egress from the nucleolus relative to WT RAG1 under V(D)J recombination-inducing conditions. However, whether CRL4^VprBP^ plays a role in subnuclear localization of RAG1 remains to be determined.

Extending the above results, we show here that ectopically-expressed murine RAG1 is turned over in a human, non-lymphoid cell line in a manner sensitive to either CRL or proteasome inhibition or loss of VprBP, suggesting widespread expression and evolutionary conservation of factors involved in RAG1 degradation. We further show that global inhibition of ubiquitylation recapitulates the effect of CRL or proteasome inhibition on RAG1 stability. Finally, we demonstrate that RAG1 association with the CRL4^VprBP^ complex is not perturbed by conditions that impair CRL activation. These data provide novel insight into the mechanistic basis of RAG1’s relatively short half-life, which, although long-recognized [3, 31], has until recently remained unexplained.

## Materials and methods

### Chemical inhibitors

MLN4924 [32] was purchased from Selleckchem and used at a final concentration of 3μM. Bortezomib was purchased from Selleckchem, and used at a final concentration of 1μM. TAK-243 [33] was purchased from ChemieTek and used at a final concentration of 0.05μM. CSN5i-3 [34] was a gift from Novartis, and was used at a final concentration of 1μM.

### Plasmids

The pEBB-MBP-FL-RAG1, cRAG1, and FL–RAG2 constructs were gifts from Michael Lieber [35]. Untagged versions of FL-RAG1 and cRAG1 were created by restriction digest and re-ligation to remove the maltose-binding protein (MBP) coding sequence. A pEBB FLAG-FLRAG1 construct was generated via a XmaI-EcoRV cassette swap between pEBB FLRAG1 and a pUltraHot construct encoding FLAG-RAG1(1–383) (Schabla and Swanson, unpublished).

### Cell transfections and protein purification

The human embryonic kidney cell line 293T (ATCC CRL-3216) was used for cell transfections. The 293T cells were maintained in 1X Dulbecco’s modified Eagle’s medium (DMEM, Corning) supplemented with 10% FBS (Atlanta Biologicals), 100 U/mL penicillin, and 0.1mg/mL streptomycin (Corning). Cells were transfected at 70% confluency as follows [36]: plasmids were mixed with polyethylenimine (PEI, 1mg/mL; Polysciences, Inc.) at a ratio of 1:3 in Dulbecco’s phosphate-buffered saline (10mM Na_2_HPO_4_, 1.8mM KH_2_PO_4_, 137mM NaCl, 2.7mM KCl) and incubated 10min at room temperature. Transfection mixes were added to cells and incubated 24h prior to harvest. For some experiments, chemical inhibitors were added at various times indicated in the figures. For RAG1 half-life experiments, cycloheximide (CHX) was added to cells for the final 4h of the transfection period, and cells were harvested at 1h intervals for analysis.

MBP-RAG fusion proteins were purified from 293T cells by affinity chromatography essentially as described [24]. Briefly, cells were washed in DPBS prior to lysis in resuspension buffer (25mM HEPES, 150mM KCl, 10mM CaCl_2_, 10% glycerol, 2mM DTT) supplemented with protease inhibitors (leupeptin, 10μM; pepstatin, 2μM; PMSF, 100μM). Cell suspensions were subjected to sonication at 23% amplitude with 10s pulses interspersed with 5s of rest for 2.5min total (Fisher Scientific Sonic Dismembranator Model 500). The resulting lysates were clarified by ultracentrifugation (22,000rpm, 40min, 4°C). Clarified cell lysate was passed over a column of amylose resin (New England Biolabs, E8021L), which was then washed with resuspension buffer prior to elution of MBP-RAGs with 10mM maltose.

FLAG (DYKDDDDK)- fusion proteins were purified by immunoaffinity chromatography as follows: cells were washed with DPBS and then lysed in 100mM HEPES, 300mM NaCl, 1mM EDTA, and 1% v/v TritonX-100. Lysates were rotated for 15min at 4°C prior to clarification by ultracentrifugation as above. Clarified lysates were added to anti-DYKDDDDK affinity gel (BioLegend) and incubated at 4°C with rotation for 2h. The gel was washed with lysis buffer (omitting EDTA and TritonX-100) and then eluted with FLAG peptide (Sigma-Aldrich) at 5mg/ml in lysis buffer containing 10% v/v glycerol.

### Western blot analysis

Cultured cells (2-4x10^6^) were incubated for 10min on ice in RIPA lysis buffer (50mM Tris, 150mM NaCl, 1% NP-40, 0.5% sodium deoxycholate, 0.1% SDS, 1mM EDTA) supplemented with 1mM sodium orthovanadate and 2% v/v protease inhibitor cocktail (Sigma-Aldrich P8340). Proteins were denatured by boiling 5min at 100°C in Laemmli sample buffer (60mM Tris-HCl pH 6.8, 2% v/v SDS, 10% glycerol, 100mM DTT, 0.02% v/v bromophenol blue) and resolved by SDS-PAGE prior to transfer onto PVDF membrane (EMD Millipore) and detection. The following primary antibodies were used: anti-VprBP (Proteintech group, 11612-I-AP), anti-RAG1 (rabbit mAb [22]; rabbit polyclonal 307 [37]), anti-β-actin (Sigma-Aldrich, A5316), anti-CUL4A (Cell Signaling Technology, #26995), anti-CUL4B (Proteintech group, 12916-1-AP), anti-MBP (New England Biolabs, E8032S), anti-UBC10 (UBE2C) (Cell Signaling Technology, #142345), and anti-UBC12 (Cell Signaling Technology, D13D7). HRP-conjugated goat-anti-rabbit or anti-mouse secondary antibodies (Cell Signaling Technology) were used to detect primary antibodies, and blots were developed using Pierce ECL2 substrate (Thermo-Fisher) and imaged using a Typhoon 9410 Variable Mode Imager (GE Healthcare). All images were generated using 457nm laser excitation with a 520/40 bandpass filter, with the PMT detector set to 600V and the image pixel size set to 100μm. All western blot signal quantification was performed using the ImageJ image processing tool (https://imagej.NIH.gov).

### Calculation of RAG1 half-life and statistical analysis

For estimation of RAG1 half-life, quantified western blot data were analyzed by linear regression, and the half-life was determined by interpolation as the time in minutes when western blot signal is half its starting value. Determination of statistical significance was done by two-way ANOVA with Bonferroni post-hoc correction. *P* values <0.05 were considered significant. All data were analyzed and plotted using GraphPad Prism.

## Results and discussion

**Fig 1:**
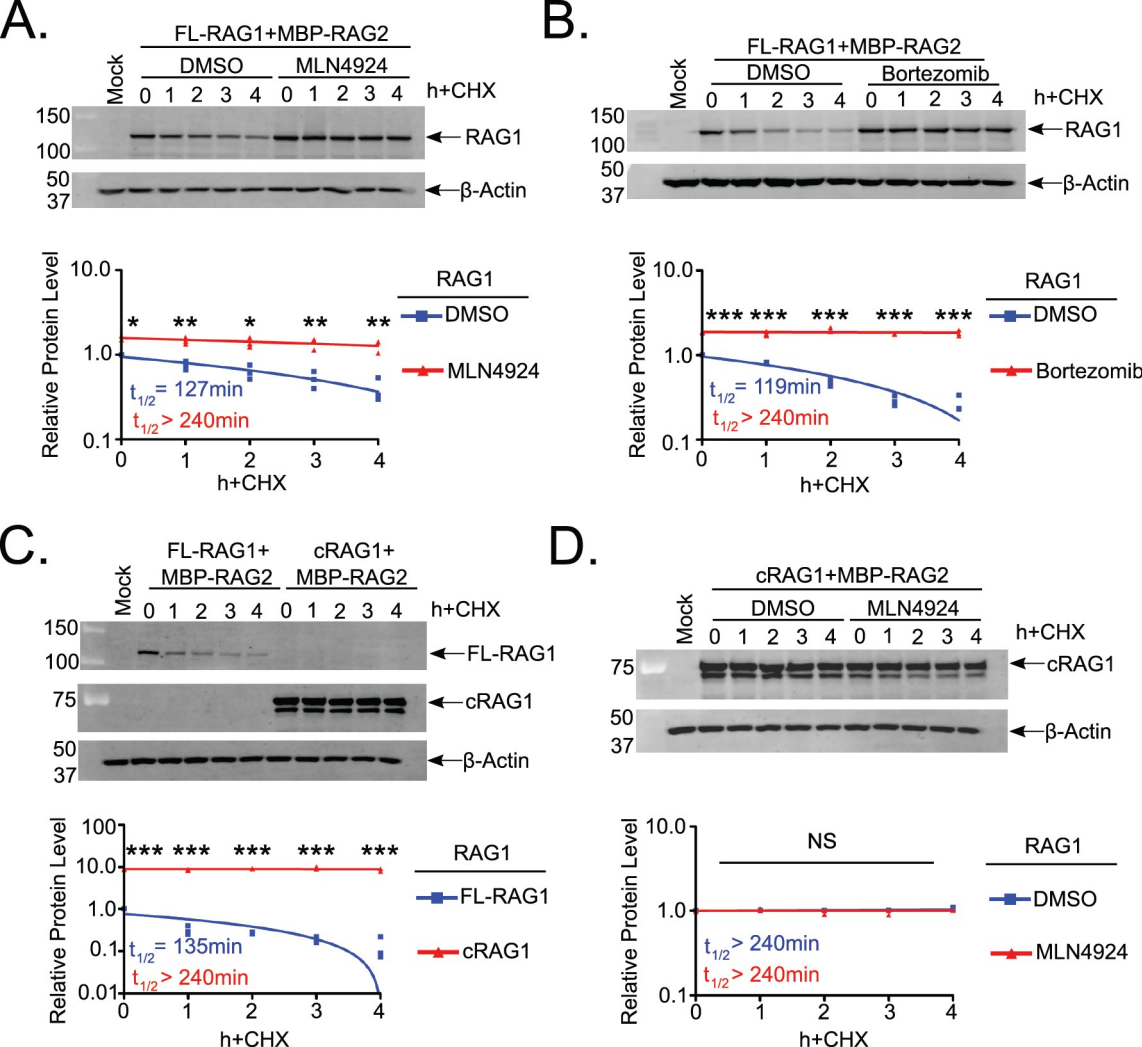
RAG1 is turned over in non-lymphoid cells in a manner dependent on N-ncRAG1.

In previous work [30], we demonstrated that VprBP (DCAF1) controls RAG1 turnover in murine B cells. To further investigate the mechanism of RAG1 turnover by VprBP, we used an experimental system in which recombinant murine full-length RAG1 and RAG2 proteins are expressed in 293T cells through transient transfection [36]. Interestingly, as was the case in murine B cells, we found that RAG1 turnover in HEK293T (hereafter called 293T) cells was dependent on both NAE and proteasome activity, as RAG1 half-life was extended by addition of MLN4924 or bortezomib, respectively (Fig 1A and 1B). This outcome suggests that the mechanism of RAG1 turnover is constitutively active, and requires factors whose expression is not restricted to lymphocytes. This is consistent with the broad expression pattern of the CRL4 components [28].

Because our previous study suggests VprBP recruits the CRL4 complex to RAG1 via ncRAG1 [20], we wished to determine whether the absence of ncRAG1 would affect RAG1 stability. To this end, we compared the turnover of FL-RAG1 and cRAG1 in 293T cells. cRAG1 was stable for the duration of cycloheximide (CHX) treatment and its basal expression level was ~10-fold higher than that of FL-RAG1 (Fig 1C). Furthermore, treating transfected cRAG1-expressing 293T cells with MLN4924 had no effect on cRAG1 stability (Fig 1D). Thus, these data are consistent with a model in which rapid RAG1 turnover is mediated by ncRAG1 association with CRL4^VprBP^ E3 Ub ligase complex. Interestingly, cRAG1 knock-in mice exhibit elevated levels of RAG1 expression compared to wild-type counterparts [9]. Though the half-life of endogenous cRAG1 was not determined, our results strongly suggest that this observation is explained by loss of interaction with CRL4 and consequent stabilization of cRAG1.

**Fig 2:**
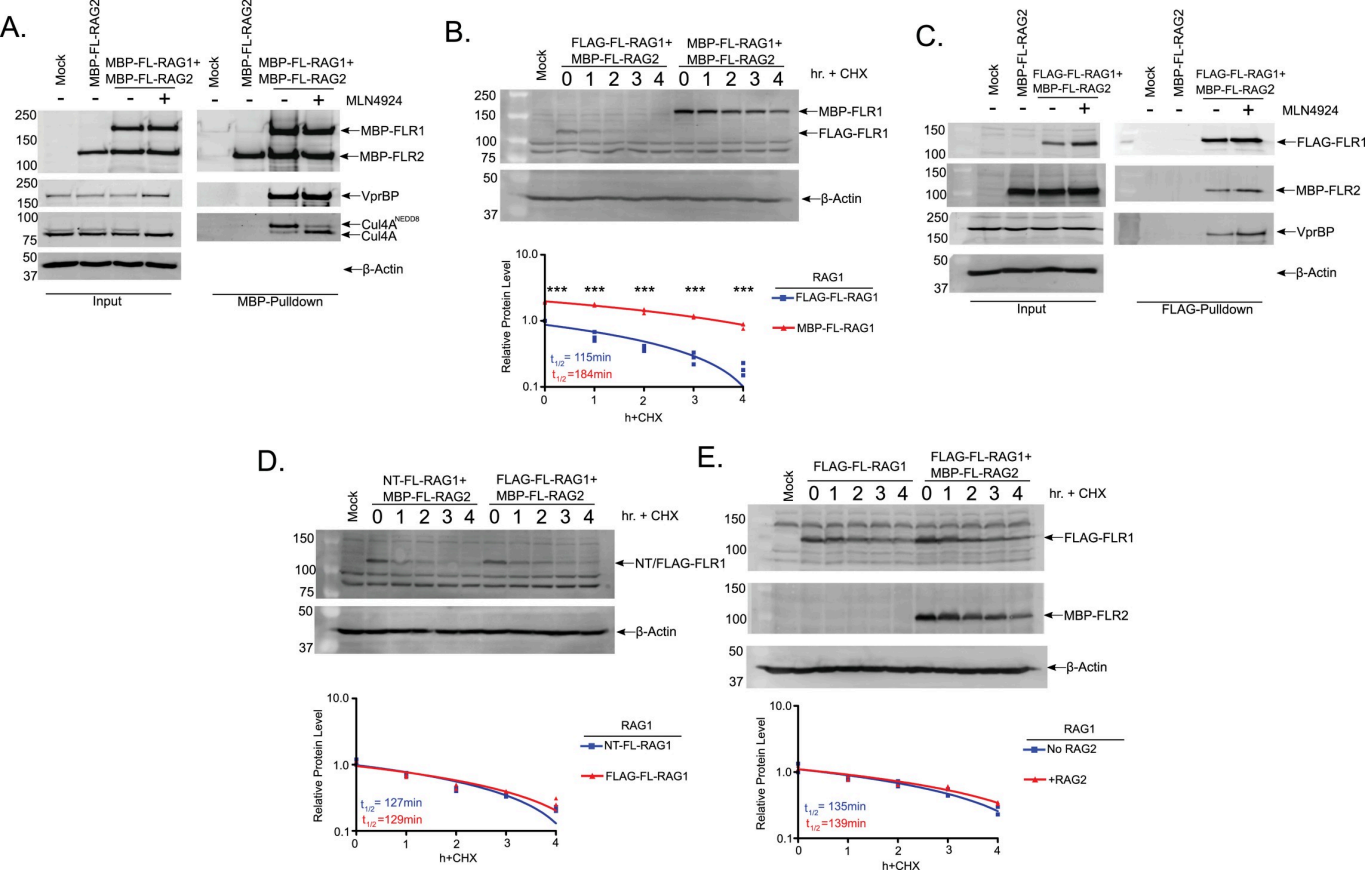
MLN4924 treatment does not block interaction between RAG1 and CRL4^VprBP^.

To explain the basis for RAG1 stabilization in 293T cells treated with MLN4924, we considered the possibility that loss of Cul4 neddylation might promote disassembly of the CRL4^VprBP^ complex, causing Cul4A to dissociate from RAG1. This scenario was suggested by previous studies showing that neddylation of the CUL subunit promotes its interaction with substrate receptors [38, 39]. To test this possibility, we purified MBP-tagged full-length RAG1 and/or RAG2 proteins (FLMR1 and FLMR2, respectively) from 293T cells treated with MLN4924 or vehicle only and probed the recovered RAG proteins for components of the CRL4^VprBP^ complex. Consistent with previous experiments [20], we detected VprBP, DDB1, and Cul4A copurifying with FLMR1/FLMR2, but not FLRM2 alone, isolated from DMSO-treated 293T cells, with the neddylated form of Cul4A predominating in the sample (Fig 2A). Interestingly, however, while MLN4924 treatment effectively prevented Cul4A neddylation, it did not promote dissociation of the CRL4^VprBP^ complex from RAG1, as non-neddylated CUL4A copurified with FLMR1/FLMR2 in approximately equal proportion to CUL4A^NEDD8^ from MLN4924- or vehicle-treated 293T cells, respectively (Fig 2A). This finding demonstrates that CUL4 neddylation is dispensable for FL-RAG1 association with the CRL4^VprBP^ E3 Ub ligase complex, but this association is not sufficient to mediate efficient RAG1 turnover.

To determine whether fusion with MBP alters the kinetics of RAG1 turnover, we directly compared the half-lives of untagged and MBP-tagged RAG1 when co-expressed in 293T cells with FLMR2 (Fig 2B). We found that MBP-tagging roughly doubles the half-life of FL-RAG1, from ~2 hr for untagged FL-RAG1 to ~3.75 hr for FLMR1 (Fig 2B). To further validate the results shown in Fig 2A, we generated an expression construct encoding an N-terminally FLAG-tagged form of FL-RAG1. FLAG-FLRAG1 was co-expressed with FLMR2 and affinity-purified from 293T cells in the presence of either vehicle or MLN4924 (Fig 2C). Similar to experiments performed with FLMR1, MLN4924 did not disrupt the interaction of CRL4 components with one another nor with FLAG-FLRAG1 (Fig 2C). Notably, treating FLAG-FLRAG1-transfected 293T cells with MLN4924 caused a more pronounced accumulation of RAG1 than FLMR1 (Fig 2C), suggesting the FLAG tag, which is only eight amino acids long, interferes less with the degradation process than MBP. Indeed, FLAG-tagged and untagged FLRAG1 exhibit a similar half-life of ~2hr (Fig 2D).

The fact that RAG2 undergoes cell cycle-dependent degradation by the SCF^Skp2^ E3 ub ligase [5, 6, 40] raises the possibility that RAG2 indirectly recruits RAG1 to the proteasome. To test this possibility, we compared the half-life of FLAG-FLRAG1 in 293T cells with or without co-transfection with FLMR2 (Fig 2E). The half-life of FLAG-FLRAG1 was ~2hr regardless of the presence of FLMR2 (Fig 2E), suggesting that RAG1 is targeted to the proteasome via a mechanism that does not require RAG2. This is consistent with our previous finding that RAG1 turnover in mouse B cells was independent of both cell cycle progression and RAG2 turnover [30].

**Fig 3:**
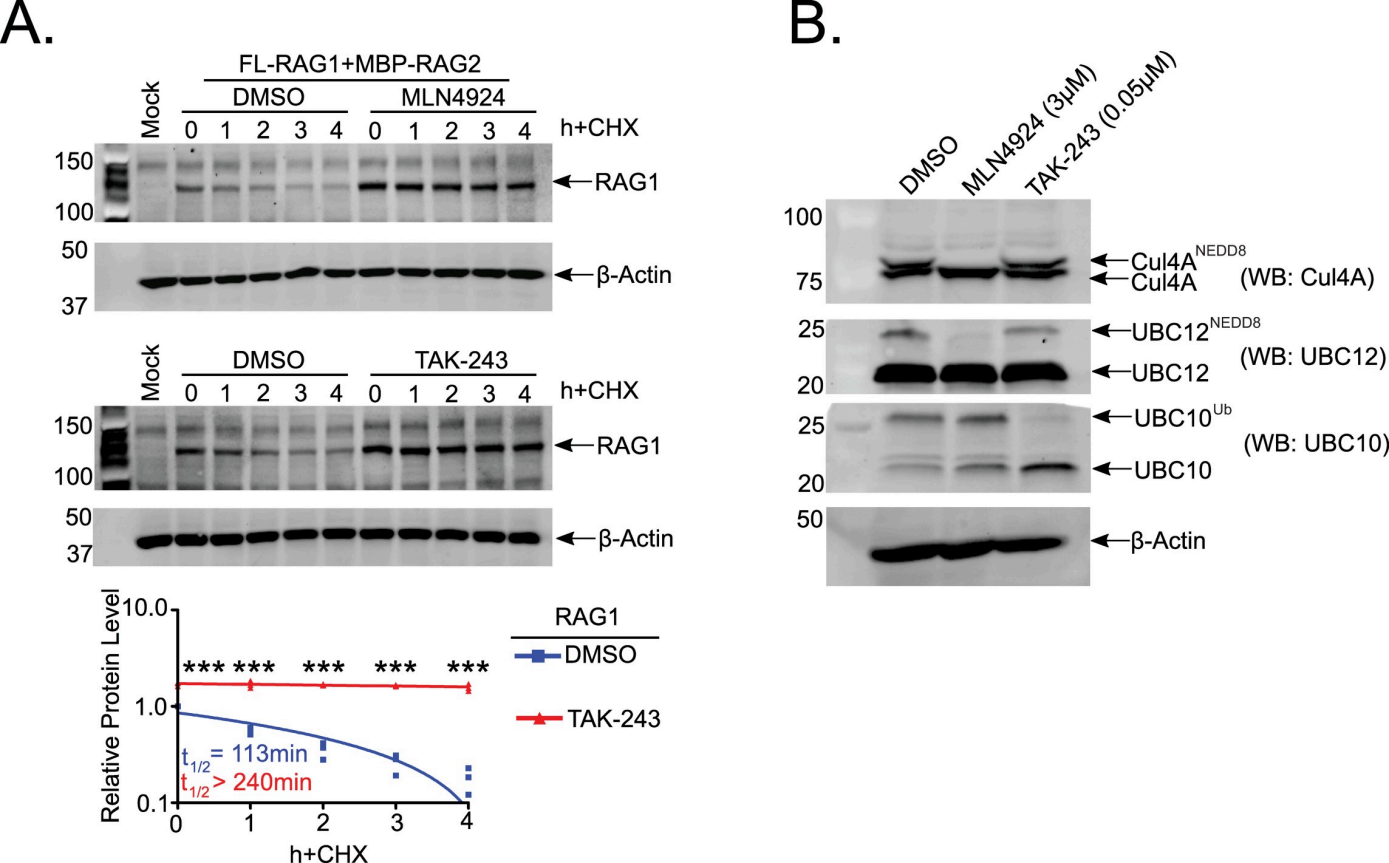
RAG1 turnover is ubiquitin-dependent.

Having established the role of CRL4^VprBP^ in RAG1 turnover, we next sought to determine whether RAG1 degradation is Ub-dependent. We treated FL-RAG-expressing 293T cells with MLN4924 or TAK-243, a recently reported UAE inhibitor [33], to globally block ubiquitylation. Similar to MLN4924 treatment, we observed that TAK-243 treatment impaired RAG1 turnover, indicating that RAG1 turnover requires Ub activation (Fig 3A). We validated the specificity of both MLN4924 and TAK-243 by analyzing lysates prepared from cells treated with vehicle or either inhibitor by non-reducing SDS-PAGE (Fig 3B). Under these conditions, the thioester bond between the UBL molecule and its corresponding E2 proteins remains intact, enabling detection of the charged E2 as a slower-migrating species in an E2-specific western blot [33]. Charging of UBC12 with NEDD8 was almost completely blocked by MLN4924, whereas this drug did not noticeably affect Ub-charging of UBC10 (Fig 3B). Conversely, TAK-243 reduced UBC10^Ub^ levels and had no effect on levels of UBC12^NEDD8^, indicating little or no cross-inhibition by these drugs at the concentrations used in the experiment (Fig 3B). In addition, neddylation of CUL4A was blocked by MLN4924, but unaffected by TAK-243 (Fig 3B). These experiments lead us to conclude that FL-RAG1 turnover is mediated by a Ub-dependent process.

Further work will be needed to determine the exact role of ubiquitylation in RAG1 turnover. The most obvious possibility is direct polyubiquitylation of RAG1 itself. However, several observations argue against this scenario. First, inhibiting proteasome activity with bortezomib does not cause detectable accumulation of any species whose SDS-PAGE migration is consistent with ubiquitylated RAG1 (see Fig 1B). Second, we have been unable to reconstitute polyubiquitylation of RAG1 by CRL4^VprBP^ in the presence of several different E2 Ub conjugating enzymes [20]. Third, previous studies demonstrate that RAG1 undergoes autoubiquitylation *in vitro* to enhance DNA cleavage activity of the RAG complex without necessarily stimulating RAG1 degradation [16, 17]. However, whether RAG1 undergoes autoubiquitylation *in vivo* and what role, if any, autoubiquitylation plays in regulating RAG activity and/or RAG1 stability remains ambiguous.

Finally, because CRL activity is promoted by cullin neddylation [26, 27], we reasoned that the RAG1 turnover kinetics would be accelerated under conditions in which Cul4 is constitutively neddylated. To test this possibility, we sought to inhibit the COP9 signalosome (CSN), a multi-subunit protein complex with endopeptidase activity. Subunit 5 (CSN5) of the complex has intrinsic metalloprotease activity and provides the catalytic function of CSN [41, 42]. In a non-redundant manner, CSN catalyzes the removal of NEDD8 from cullin proteins, which abrogates the E3 ligase activity of CRLs, and in some cases, promotes their disassembly [43, 44]. Recently, a small-molecule inhibitor of CSN5, called CSN5i-3, was developed and reported to block deneddylation of CUL1-4 in HCT116 cells, trapping their respective CRL complexes in a constitutively active state [34].

**Fig 4:**
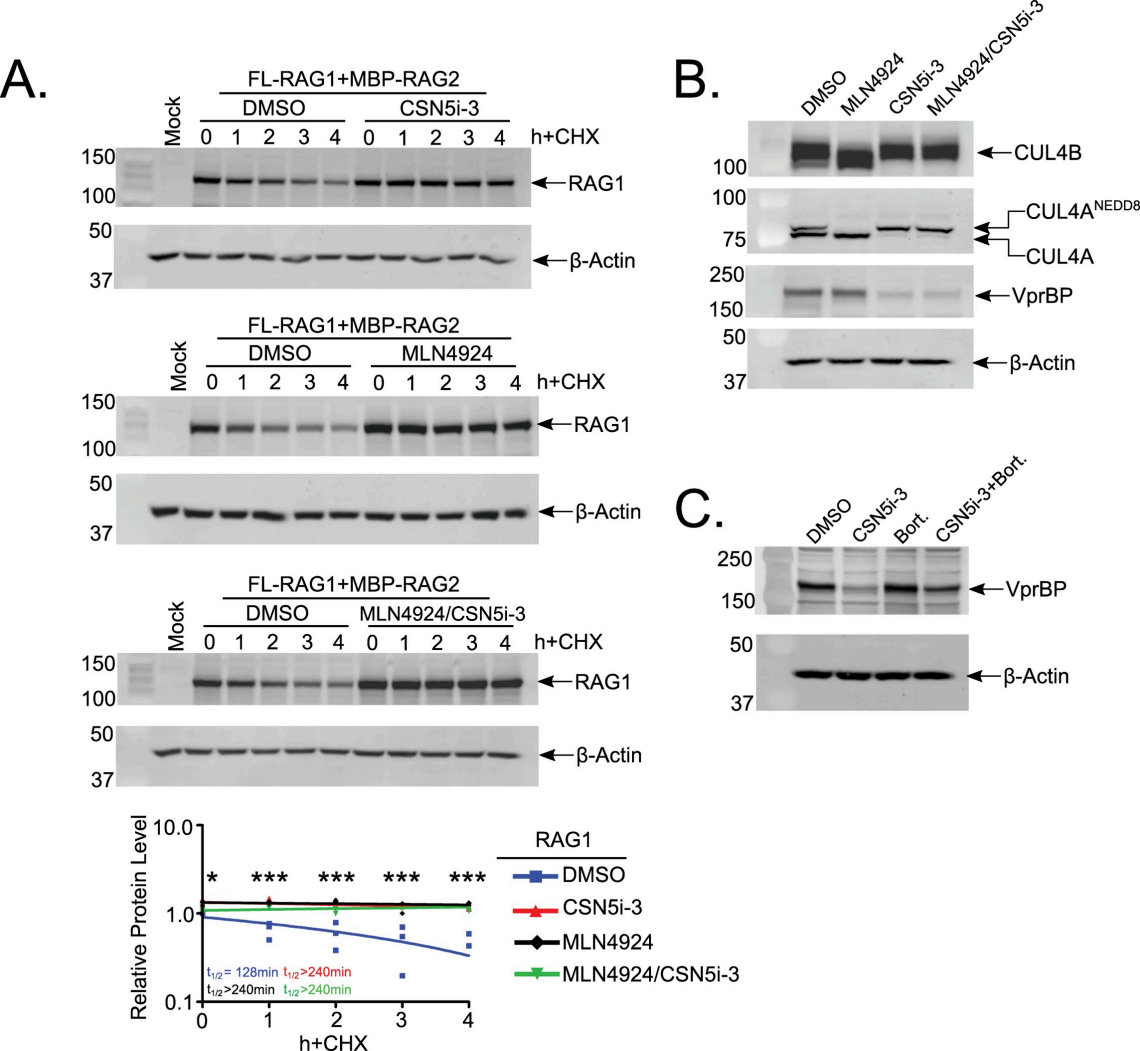
Inhibition of CSN5 stabilizes RAG1.

We treated 293T cells transfected with FL-RAG constructs with CSN5i-3 for 20h prior to the adding of DMSO or MLN4924 and CHX for 4h. Unexpectedly, we found that CSN5i-3 inhibited RAG1 turnover when added to cells alone (Fig 4A, upper panel; treatment with MLN4924 alone shown for comparison, middle panel). RAG1 turnover exhibited nearly identical turnover kinetics when cells were treated with both inhibitors (Fig 4A, lower panel). Western blotting revealed predominance of the neddylated species of CUL4A and CUL4B in CSN5i-3-treated cells, and that treatment with MLN4924 did not affect neddylation levels in the presence CSN5i-3 (Fig 4B). We also found that VprBP levels were reduced in the presence of CSN5i-3 (Fig 4B). Interestingly, a previous study showed that, in the presence of CSN5i-3, constitutively active CRL complexes polyubiquitylate their substrate-adaptor proteins, targeting them for proteasomal degradation [34]. We therefore surmise that the RAG1 stabilization in the presence of CSN5i-3 likely results from loss of VprBP. Consistent with this possibility, simultaneous treatment of 293T cells with CSN5i-3 and bortezomib partially rescues VprBP levels relative to treatment with CSN5i-3 alone (Fig 4C). Although this experiment did not allow us to determine whether constitutive activation of CRL4 accelerates RAG1 turnover, it recapitulated in human, non-lymphoid cells our finding in murine B cells that VprBP is required for RAG1 turnover.

## Conclusion

Despite RAG expression being restricted to developing lymphocytes *in vivo*, we show here that RAG1 is subject to turnover in 293T cells via a mechanism that has similar requirements for NAE and proteasome activity, as well as ubiquitin and VprBP. Turnover of murine RAG1 in a human cell line implies evolutionary conservation of the degradation pathway. Further work will be required to determine the substrate(s) targeted by CRL4^VprBP^ to recruit RAG1 to the proteasome.

## Supporting information

S1 Raw images Uncropped images for Figures. (PDF)
