# Supplementary material for: The CRL4VPRBP(DCAF1) E3 ubiquitin ligase directs constitutive RAG1 degradation in a non-lymphoid cell line
Source: PLoS One. 2021 Oct 14;16(10):e0258683. doi: 10.1371/journal.pone.0258683 (PMC8516306; doi:10.1371/journal.pone.0258683)
Supplement: S1 Raw images — (PDF) [file pone.0258683.s001.pdf]

Fig 1A

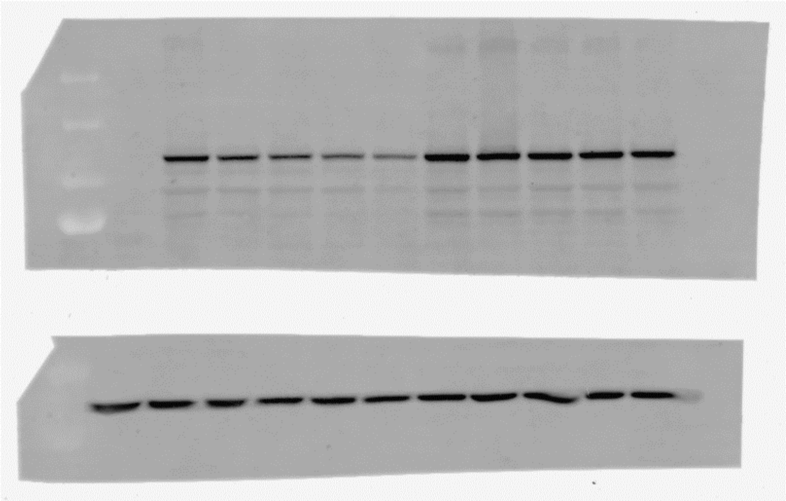

Fig 1B

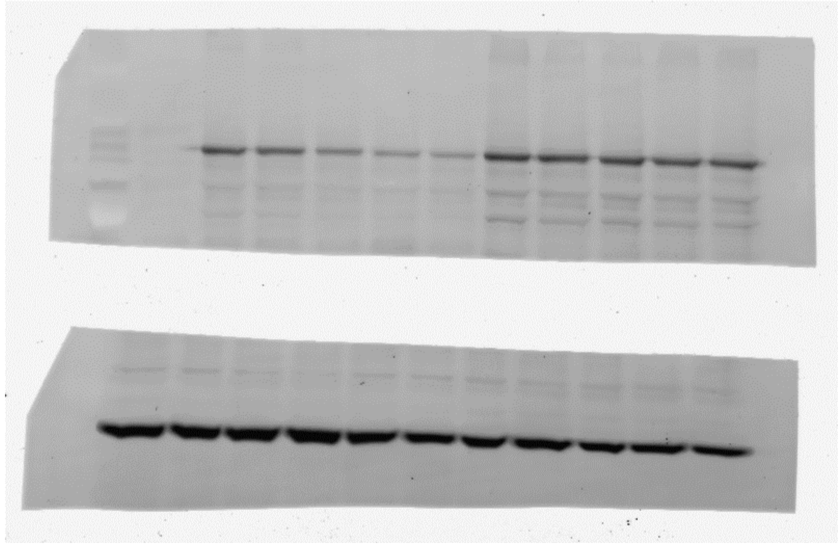

Fig 1C

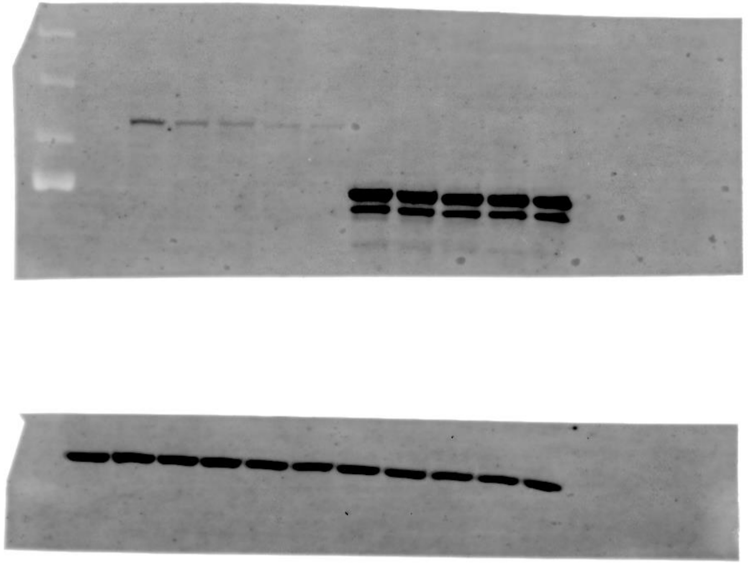

Fig 1D

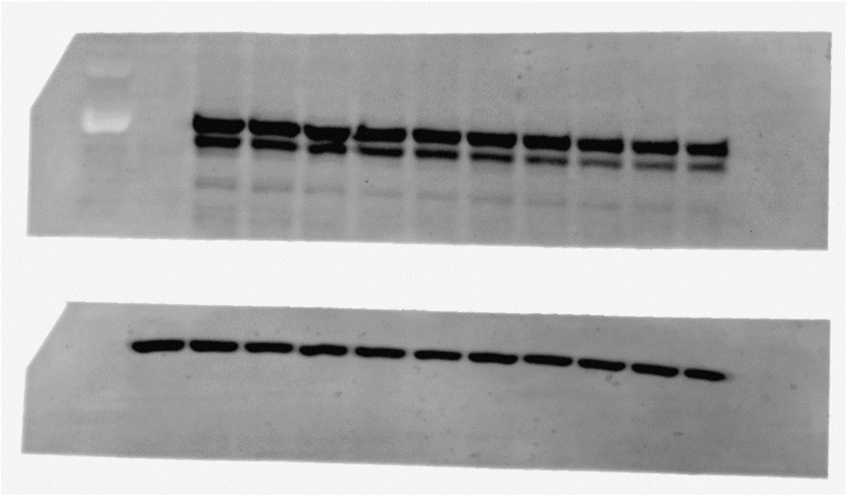

Fig. 1 Uncropped blots

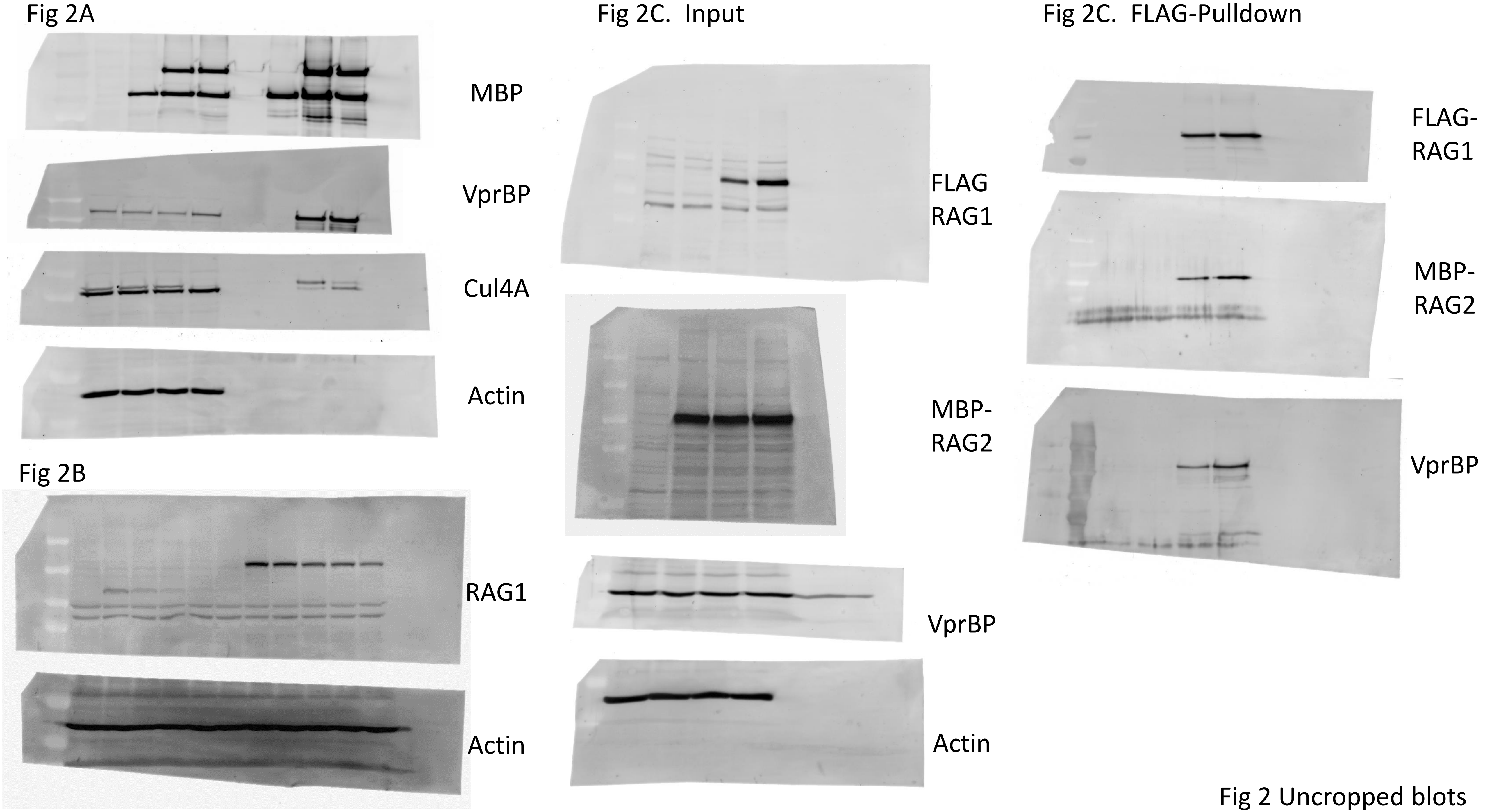

Fig 2 Uncropped blots

Fig 2D

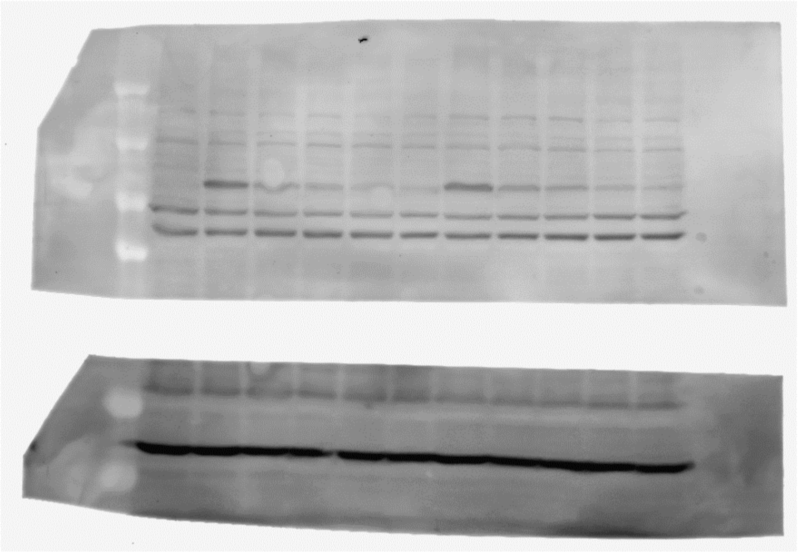

Fig 2E

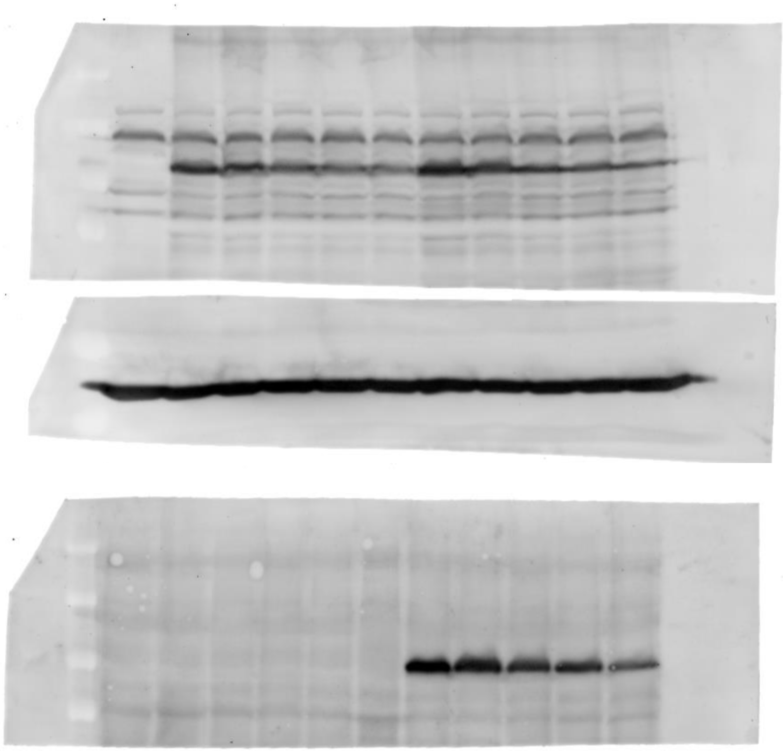

Fig. 2 Uncropped blots

Fig 3A Top Panels

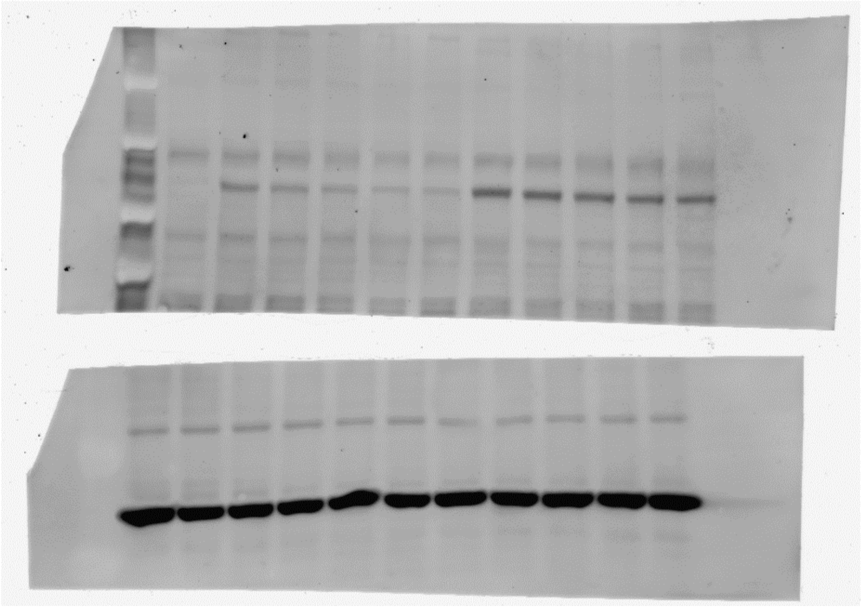

Fig 3A Bottom Panels

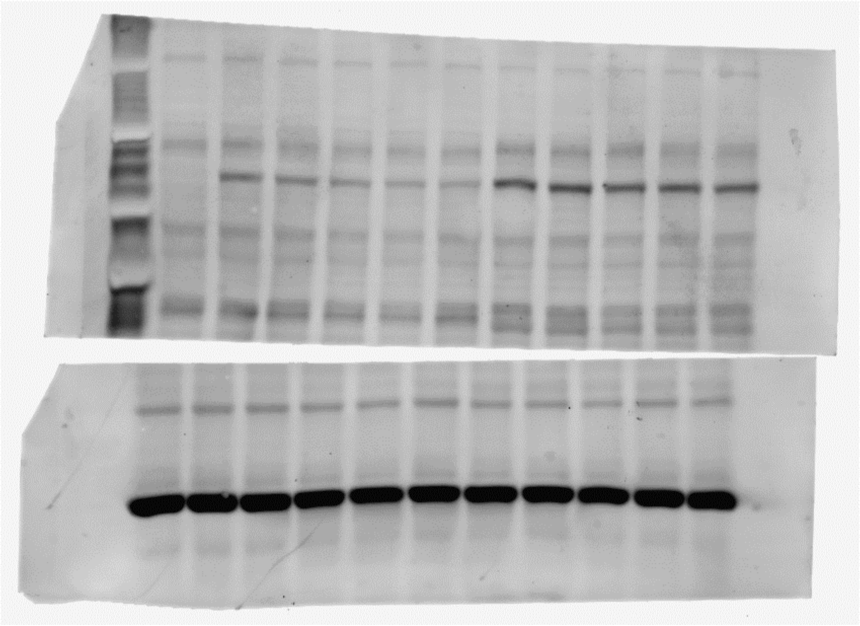

Fig. 3B

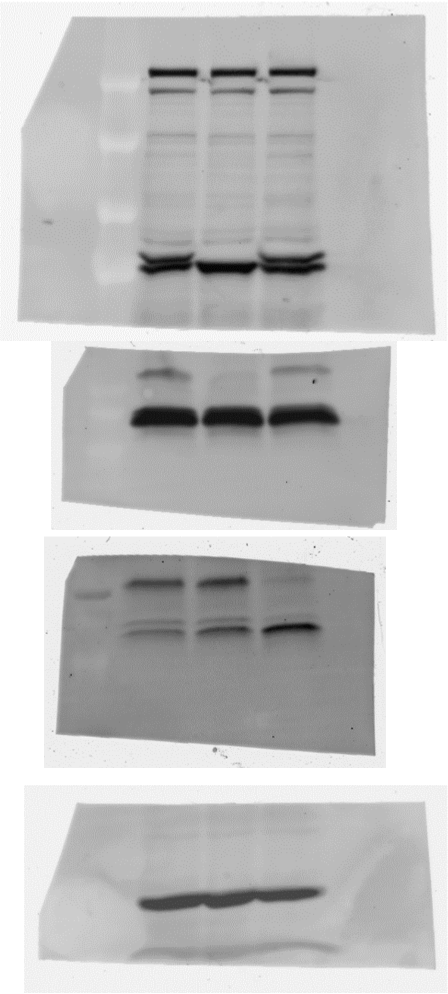

Cul4A

UBC12

UBC10

Actin

Fig. 3 Uncropped blots

Fig. 4A Top Panels

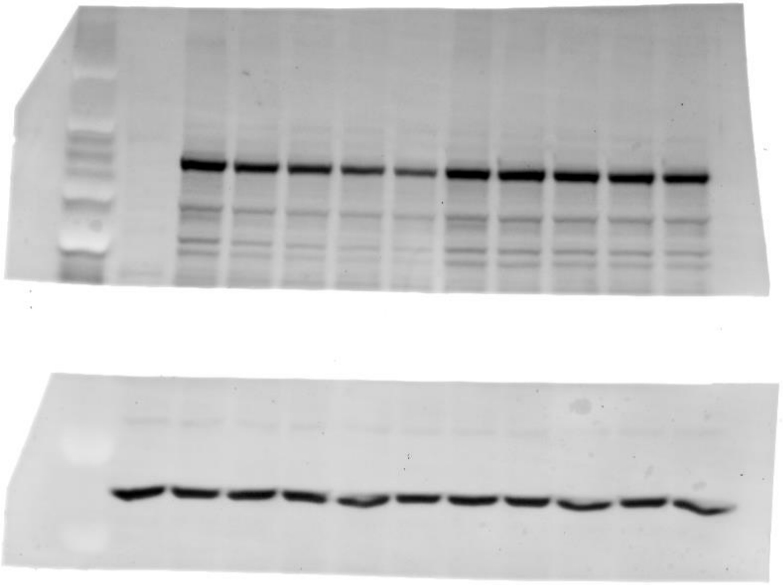

Fig. 4A Middle Panels

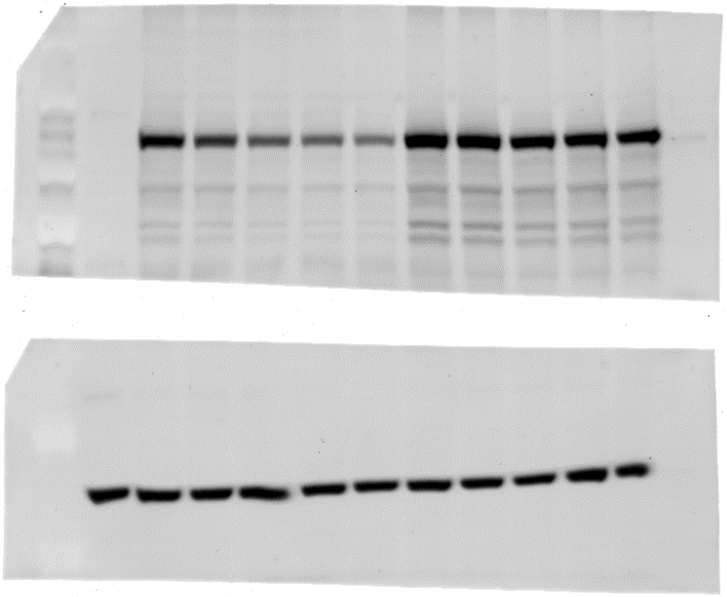

Fig. 4A Bottom Panels

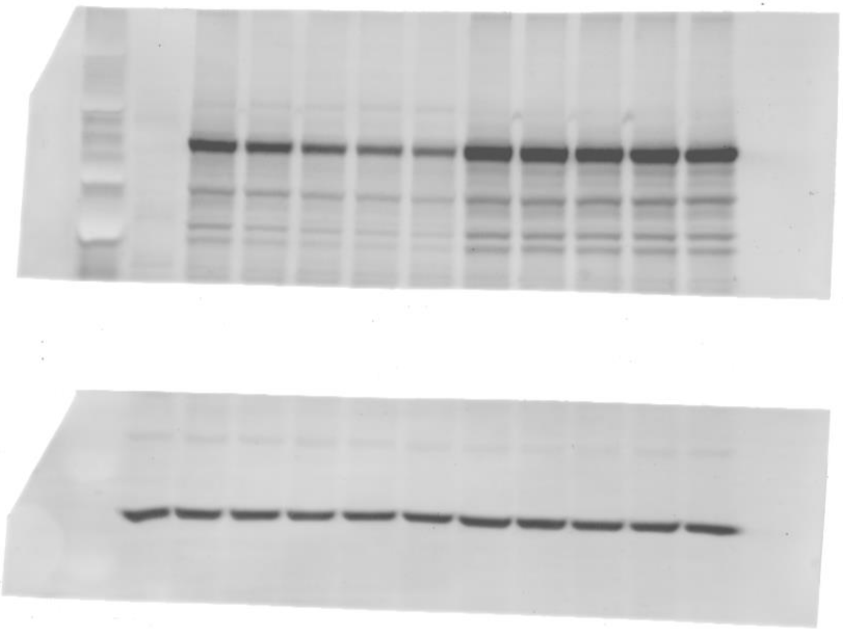

Fig. 4 Uncropped blots

Fig. 4B

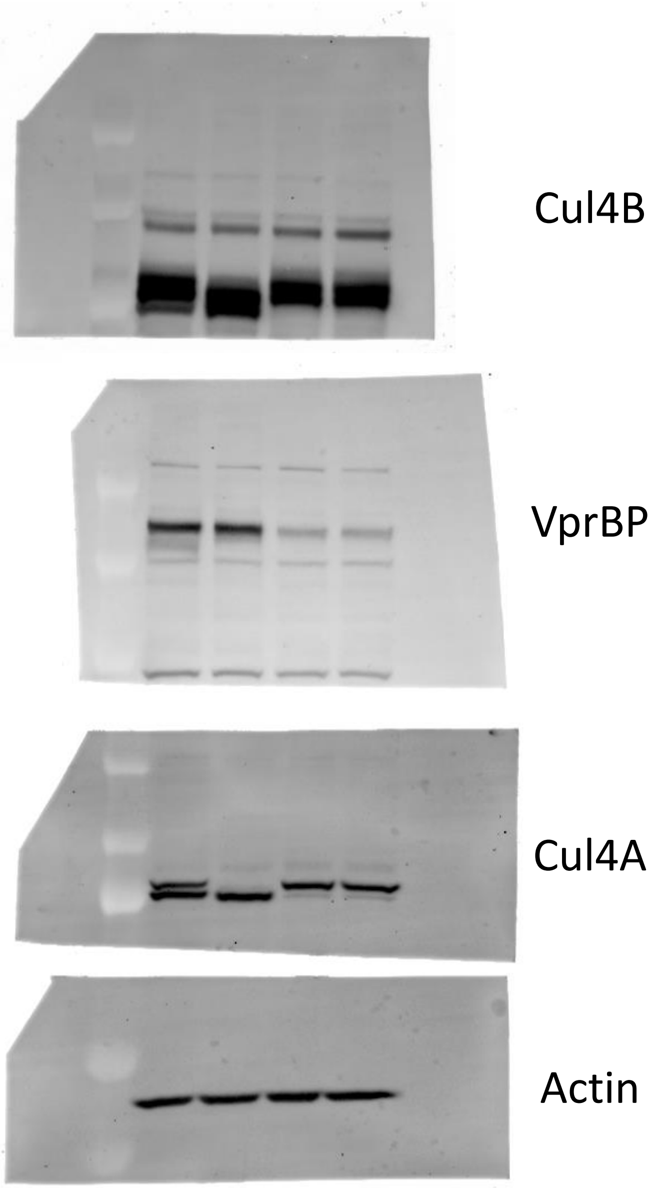

Fig. 4C

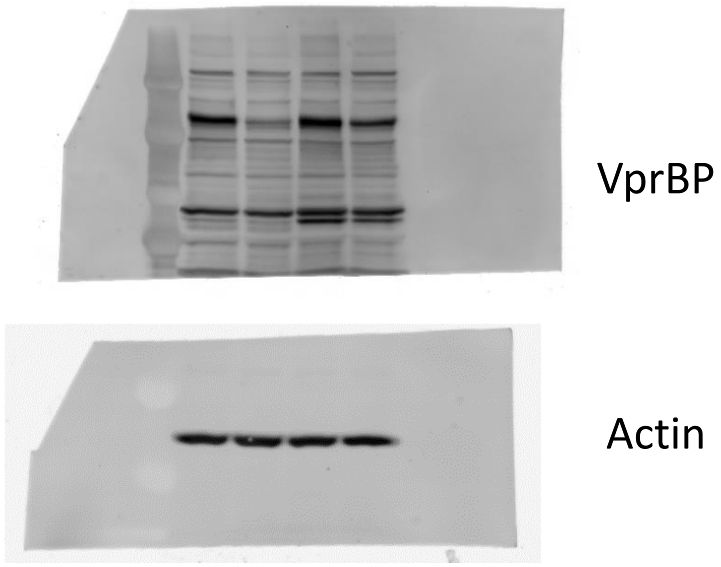

Fig. 4 Uncropped blots
